# Evaluation of the 2020 American Urological Association Microscopic Hematuria Guidelines in Clinical Practice: Retrospective Chart Review Analysis

**DOI:** 10.2196/75929

**Published:** 2025-12-04

**Authors:** Dominique Munroe, James O'Keefe, Danyang Wang, Miranda A Moore

**Affiliations:** 1 Department of Family Medicine, Atrium Health Navicent, 777 Hemlock St, Macon, GA, 31201, United States, 1 4705109235; 2 Department of Medicine, Emory University, Atlanta, GA, United States; 3 Department of Family and Preventive Medicine, Emory University, Atlanta, GA, United States

**Keywords:** hematuria, cystoscopy, guideline, risk stratification, urinalysis

## Abstract

**Background:**

Hematuria is one of the most common urologic diseases seen within clinical practice, with a prevalence range of 1.7%‐31.1%. In 2020, American Urological Association (AUA) guidelines were revised and recommend that following initial evaluation, clinicians should categorize patients into three tiers (low risk, intermediate risk, and high risk) based on various factors. Recent literature has shown that the AUA guidelines have high clinical utility when compared to other international guidelines such as those outlined by the Hematuria Risk Index, Canadian Urological Association, and Kaiser Permanente; however, this guideline remains unvalidated among the population of “well adults” within the United States.

**Objective:**

We used a retrospective study design to evaluate data abstracted from the electronic medical records of patients seen in the Emory Healthcare Executive Health Clinic from September 29, 2017, to January 29, 2021, to investigate the utility of risk stratification as a tool for clinical decision-making.

**Methods:**

According to the AUA risk stratification system, patients were stratified into low-risk and intermediate-risk/high-risk groups based on sex, age, smoking history, history of gross hematuria, and red blood cells/high-powered field. The frequencies and percentages of different causes of hematuria across the three risk strata were reported.

**Results:**

Of the 882 instances of red blood cells in urine (URBC) ≥3, a total of 368 (41.72%) underwent a repeat analysis within a 6-month time span, 184 (20.86%) within a 12-month time span, and 330 (37.41%) at >12 months. Instances of a URBC <3 (N=1643) were more likely to have no urologic diagnosis—1503 (91.48%) in comparison to 633 (76.27%) for those instances with a URBC >3 (N=830). Ultimately, 23 (100%) participants in the low-risk group had no urologic diagnosis after urinalysis versus 608 (75.62%) in the intermediate-risk/high-risk group (N=804).

**Conclusions:**

We found a need for a greater focus on monitoring elevated URBC counts, in accordance with clinical guidelines for managing hematuria in low-risk patients. Future research should examine the impact of risk stratification on clinical decisions and access to care, especially in underserved populations. It should also assess how the new AUA guidelines affect physician referral patterns and explore real-world implementation challenges and facilitators.

## Introduction

The presence of blood in the urine (hematuria) may indicate the presence of underlying diseases such as infections, kidney stones, or cancer of the urinary system [1,2]. Hematuria is one of the most common urologic diseases, accounting for over 20% of urological evaluations [3]. Other studies have demonstrated a prevalence range between 2.4% and 3.1% among healthy volunteers [4]. The two categories of hematuria observed in the literature are gross (or visible) hematuria and microscopic hematuria (MH). There is general consensus that the presence of gross hematuria necessitates a complete evaluation for urologic cancers [4-6], whereas MH is often an incidental finding, and the medical community has not adhered to universal diagnostic evaluation recommendations [7,8].

In 2012, the American Urological Association (AUA) created a recommendation to guide the medical care of patients presenting with MH, recommending further medical testing for all patients over age 35 who do not have a benign cause identified (eg, menstrual period, vigorous exercise, or infection) [4]. Contrary to AUA recommendations at that time, other professional organizations created guidelines that recommend against screening for adults at low cancer risk [2,9]. In 2020, these AUA guidelines were revised [5]. Among the revisions was the recommendation that following initial evaluation, clinicians should categorize patients into 3 tiers (low risk, intermediate risk, and high risk) based on various factors. Patients who were considered intermediate risk or high risk were recommended to undergo further evaluation, while those who were low risk were recommended to undergo a repeat urinalysis within 6 months or undergo other testing.

Use of the 2020 AUA guidelines has been shown to be a cost-effective alternative to more invasive testing through its unique ability to prioritize more costly and invasive workups for patients at greatest risk [8,10,11].

However, despite its utility, this guideline remains unvalidated. The goal of this study is to add to the growing literature surrounding the utility of the AUA index, further guiding clinical practice in the population of “well adults” while improving evaluation rates for high-risk patients and decreasing rates of unnecessary testing for low-risk patients.

## Methods

### Study Design

Data were abstracted from the electronic medical records of patients seen in the Emory Healthcare Executive Health Clinic from September 29, 2017, to January 29, 2021. The Executive Health Clinic’s standard annual physical includes a routine urine collection, thus providing a population of patients with sufficient observations to adequately analyze the impact of the AUA guideline changes. We collected data on demographics, smoking history, results of urinalysis, instances of follow-up imaging or cystoscopy after urinalysis, history of gross hematuria, and urologic diagnoses of the patients.

The results of urinalysis were categorized into four groups according to degree of hematuria: 0‐2, 3‐10, 11‐25, and >25 red blood cells (RBC)/high-powered field (HPF). For each group, the instances and percentages of initial repeat urinalysis, imaging, and cystoscopy within 6 months or 12 months or after 12 months were reported. The original four groups were further consolidated into two categories—URBC (red blood cells in urine) <3 and URBC ≥3—to allow more streamlined and interpretable evaluation. Additionally, this method also ensured sufficient sample size within each category to support a robust statistical analysis.

Although the AUA risk stratification system (Textbox 1) [5] stratifies patients into low-, intermediate-, and high-risk groups based on sex, age, smoking history, history of gross hematuria, and RBC/HPF, the main distinction between intermediate- and high-risk patients is the number of smoking pack-years. Unfortunately, our electronic health record does not provide adequate information to determine the number of pack years for our patients. Thus, we combined the intermediate- and high-risk groups for our analysis. Patients with any of the following were classified as being in the intermediate-/high-risk group: women age ≥50 years or men age ≥40 years; ≥10 pack-years smoking; >10 RBC/HPF on one urinalysis; history of gross hematuria; and previously low-risk with no prior evaluation and ≥3 RBC/HPF on repeat urinalysis. The causes of hematuria were categorized as stones, infections, glomerular noninfections, benign prostate diseases, other urologic benign diseases, malignant bladder diseases, malignant kidney diseases, malignant prostate diseases, malignant ureter diseases, other urologic malignant diseases, and nonurologic diseases. The frequencies and percentages of different causes of hematuria across the 4 hematuria degrees or risk strata were also reported. To calculate an effect size for final diagnosis by initial URBC value and risk stratification, we computed Cohen *h* to quantify the difference between two proportions.

A new urine collection technique (midstream urine collection) for males has been applied since November 21, 2019. The impact of this technique change on urinalysis results among males versus females was examined using 2-tailed *χ*^2^ tests, 2, with *P*<.05 indicating statistical significance.

Textbox 1. **American Urological Association risk stratification guidelines 2020.**

**Low (patient meets all criteria)**

- Women age <50 years or men age <40 years
- Never smoker or <10 pack-years
- 3‐10 red blood cells (RBC)/high-powered field (HPF) on a single urinalysis
- No risk factors for urothelial cancer

**Intermediate (patient meets any one of these criteria)**

- Women age 50‐59 years or men age 40‐59 years
- 10‐30 pack-years
- 11‐25 RBC/HPF on a single urinalysis
- Low-risk patient with no prior evaluation and 3‐10 RBC/HPF on repeat urinalysis
- Additional risk factors for urothelial cancer

**High (patient meets any one of these criteria)**

- >30 pack-years
- Women or men age ≥60 years
- >25 RBC/HPF on a single urinalysis
- History of gross hematuria

### Ethical Considerations

Emory University’s institutional review board approved this retrospective study in January 2022 (protocol number 2022P004116). All data analysis was performed using StataSE 14 (StataCorp LLC) [12].

## Results

A total of 2677 unique patients gave 5072 urine samples at the Emory Healthcare Executive Health Clinic from September 29, 2017, to January 29, 2021. The mean age of the cohort was 58.07 years, with 1770 (73.9%) participants identifying as male and 625 (26.1%) identifying as female.

Overall, 3700 (72.97%) instances of a URBC count of <3 were observed within this cohort compared to 1372 (27.05%) instances of URBC ≥3 (Table 1). Of the total 5072 instances observed, 2677 were unique patients, whereas the remaining instances were accounted for by patients who appeared multiple times within the data.

**Table 1.** Initial instances of red blood cells in urine. There were 2677 unique patients seen in the Emory Healthcare Executive Health Clinic from September 29, 2017, to January 29, 2021 (N=5072 urine samples).

Red blood cells in urine count	Value, n	Percentage
<3	3700	72.95
≥3	1372	27.05

Of the 1795 instances of URBC <3, there were 287 (15.97%) that were offered a repeat within 6 months (Table 2), 487 (27.13%) within 12 months, and 1021 (56.88%) more than 12 months since the initial urinalysis. In comparison, 882 of the instances of URBC ≥3 underwent a repeat analysis, with 368 (41.72%) being referred within a 6-month time span, 184 (20.86%) within 12 months, and 330 (37.41%) more than 12 months later.

**Table 2.** Initial urinalysis result and referral timeline. There were 2677 unique patients seen in the Emory Healthcare Executive Health Clinic from September 29, 2017, to January 29, 2021.

	URBC^a^ <3 (n=1795)		URBC ≥3 (n=882)	
	Value, n	Mean %	Value, n	Mean %
Within 6 months	287	15.99	368	41.72
Within 12 months	487	27.12	184	20.86
Over 12 months	1021	56.88	330	37.41

a URBC: red blood cells in urine.

Regarding referral patterns following initial urinalysis results, those with URBC ≥3 were referred for follow-up imaging and procedures 40.21% (421/1047) of the time in comparison to 30.38% (371/1221) for those with URBC <3 (Table 3). Instances with URBC <3 were responsible for 20% (21/105) of cystoscopies and 52.78% (171/324) of any imaging within the 6-month time frame; 25% (32/130) of cystoscopies and 55% (219/396) of any imaging in the <12-month time frame; and 63% (51/81) of cystoscopies and 66% (144/219) of any imaging within the >12-month time frame (Table 4).

**Table 3.** Number (%) of initial screenings via imaging and procedures after urinalysis by timing and urinalysis results. There were 2677 unique patients seen in the Emory Healthcare Executive Health Clinic from September 29, 2017, to January 29, 2021.

	URBC^a^ <3 (n=1221)		URBC ≥3 (n=1047)	
	Values, n	Percentage	Values, n	Percentage
Within 6 months				
Any imaging	171	14	153	14.61
CT^b^	89	7.29	97	9.26
MRI^c^	26	2.13	18	1.72
USG^d^	56	4.59	38	3.63
Cystoscopy	21	1.72	84	8.02
Any imaging + cystoscopy	8	0.66	31	2.96
Within 12 months				
Any imaging	219	17.94	177	16.91
CT	118	9.66	110	10.51
MRI	37	3.03	23	2.2
USG	64	5.24	44	4.2
Cystoscopy	32	2.62	98	9.36
Any imaging + cystoscopy	20	1.64	38	3.63
Over 12 months				
Any imaging	144	11.79	75	7.16
CT	72	5.90	39	3.72
MRI	32	2.62	15	1.43
USG	40	3.28	21	2.01
Cystoscopy	51	4.18	30	2.87
Any imaging + cystoscopy	21	1.72	13	1.24

a URBC: red blood cells in urine.

b CT: computed tomography.

c MRI: magnetic resonance imaging.

d USG: ultrasonography.

**Table 4.** Number (%) of diagnoses of patients after urinalysis by URBC^a^ category. There were 2677 unique patients seen in the Emory Healthcare Executive Health Clinic from September 29, 2017, to January 29, 2021.^b^

Final diagnosis	URBC <3 (n=1643)		URBC ≥3 (n=830)	
	Values	Percentage	Values	Percentage
No urologic diagnosis	1503	91.48	633	76.27
Stones	24	1.46	42	5.06
Infection	15	0.91	28	3.37
Glomerular noninfection	1	0.06	2	0.24
Benign prostate	45	2.74	59	7.11
Benign urologic other^c^	32	1.95	40	4.82
Malignant bladder	1	0.06	2	0.24
Malignant kidney	4	0.24	5	0.60
Malignant prostate	18	1.10	15	1.81
Malignant ureter	0	0.00	2	0.24
Malignant urologic other^c^	0	0.00	1	0.12

a URBC: red blood cells in urine.

b Cohen *h*=0.425.

c Site not specified.

Instances of URBC <3 were more likely to have no urologic diagnosis (1503/1643, 91.48%) compared to those instances with a URBC >3 (633/830, 76.27%; Table 4). The Cohen *h* of 0.425 indicates that there is a medium-sized difference in the proportion of patients with no urologic diagnosis between those with URBC <3 and those with URBC >3. Of urologic problems identified for instances of URBC <3, the most common diagnoses were benign prostate, benign urologic (site not specified), and stones (2.74% vs 1.95% vs 1.46%, respectively; Table 4). Further stratification of instances with ≥3 RBC/HPF revealed that 23 instances would have been classified as low risk and 804 (97%) as intermediate/high risk (Table 5). Most notably, in the low-risk instances, 100% had no urologic diagnosis after urinalysis versus 75.62% in the intermediate-/high-risk group. The Cohen *h* of 1.033 indicates that there is a large-sized difference in the proportion of patients with no urologic diagnosis between those with low risk and those with high risk.

**Table 5.** Number (%) of diagnoses of patients after urinalysis by risk stratification^a^.

	Low risk (n=23)		Intermediate/high risk (n=804)	
	Value, n	Percentage	Value, n	Percentage
No urologic diagnosis	23	100	608	75.62
Stones	0	0	42	5.22
Infection	0	0	27	3.36
Glomerular noninfection	0	0	2	0.25
Benign prostate	0	0	59	7.34
Benign urologic other^a^	0	0	40	4.98
Malignant bladder	0	0	3	0.37
Malignant kidney	0	0	5	0.62
Malignant prostate	0	0	15	1.87
Malignant ureter	0	0	2	0.25
Malignant urologic other^b^	0	0	1	0.12

a 781 unique patients with ≥3 red blood cells/high-powered field on at least one urinalysis were stratified. Cohen *h*=1.033.

b Site not specified.

Significant differences were observed in the detection of URBC <3 RBC/HPF in males after implementation of the new urine collection technique (Table 6). We found that of 2763 male samples collected, 1812 (71.14%) males had a URBC <3 after the new technique in comparison to 951 (92.98%) after the new technique (*P*<.001). Of the 930 female samples collected, 586 (65.4%) had a URBC <3 before the technique change versus 351 (72.67%) after the technique change (*P*=.006; Table 6).

**Table 6.** Differences in frequencies of red blood cells in urine ≥3 before versus after the new collection technique for males.

	Before technique change (n=3443)		After technique change (n=1629)		Before versus after difference *P *value
	Value, n	Percentage	Value, n	Percentage	
Male (n=2763)					
<3 RBC/HPF^a^	1812	71.14	951	82.98	<.001
≥3 RBC/HPF	735	28.86	195	17.02	
Female (n=930)					
<3 RBC/HPF	586	65.4	351	72.67	.006
≥3 RBC/HPF	310	34.6	132	27.33	

a RBC/HPF: red blood cells/high-powered field.

## Discussion

Our results showing most patients with URBC ≥3 underwent repeat testing indicates a stronger emphasis on monitoring elevated URBC counts, which aligns with the clinical guideline for managing hematuria in low-risk patients [1]. However, the extended follow-up period observed raises questions concerning the adherence to management guidelines, which recommend earlier repeat testing to rule out significant urologic pathology [5]. These findings are consistent with research showing a lack of consistency with the evaluation of hematuria across multiple health care settings; it has been observed that physicians often do not refer for appropriate urologic investigation [7,13]. Furthermore, when looking at a large managed-care organization, out of the 456,674 patients diagnosed with MH, 1.7% were seen by a urologist [14]. Even more troubling, disparities surrounding urologic referral rates have been shown for age, sex, and race [13,15-18]. These disparities indicate the need for nationally recognized algorithms such as the AUA guidelines. When used correctly, these clinical guidelines have been shown to result in standardized referral patterns and timelines across diverse practice settings, reducing variability and promoting consistent patterns in patient care [19-22].

The use of risk factors for bladder cancer such as age, male, sex, smoking, and gross hematuria as stratification tools has been associated with increased incidence in various studies [14,23]. Our results, which showed that patients who were placed in the low-risk group with URBC ≥3 had no subsequent urologic diagnosis in comparison to the intermediate-risk and high-risk group, further provide support for the use of these factors as the foundation of clinical decision-making.

Our low incidence of cancer after urinalysis is consistent with previous research showing low rates of malignancy detection after evaluation of MH patients [23,24] and further strengthens the argument against unnecessary imaging and cystoscopies. Unnecessary invasive procedures can have potential side effects such as allergic reactions, increased cost, and radiation-induced malignancy [23,25,26]. Refining the MH guidelines reduces unnecessary invasive procedures in low-risk patients and avoids the potential harms of using unnecessary contrast-enhanced imaging.

The implementation of the midstream urine collection technique leading to a decrease of URBC >3 suggests that the technique change may be more effective in reducing false-positive findings of hematuria in males; however, as no adjustments for potential confounders were made, these results should be interpreted with caution. Although the improvement in females was statistically significant, the effect size was smaller, indicating that other factors may influence URBC levels in women or that the technique may be less effective within this population. This is consistent with research that showed that catheterization may be a better method to diagnose and screen for MH in women [27]. For example, a study showed that significantly fewer red blood and squamous epithelial cells were found in catheterized urinalysis samples. Furthermore, when applied as part of the criteria for a properly collected sample, the positive predictive value of referral urinalysis increased by 22.7% for true MH [27].

One of the primary limitations of our study was its retrospective design, which inherently restricts the ability to draw causal conclusions; its reliance on preexisting data may introduce biases [28].

Our patient population, consisting exclusively of individuals receiving care through the Executive Health Clinic, possesses substantial advantages such as reliable access to comprehensive preventive services, follow-up visits, high-quality private insurance, greater health literacy, and fewer barriers to care. In contrast, the broader and more diverse US patient population—many of whom may face significant health disparities and systemic barriers to care—may experience markedly different health trajectories. As a result, the associations observed in our cohort may be confounded by these advantages, potentially creating the illusion of stronger relationships between risk factors and outcomes that may be observed when examined in less advantaged populations.

As we needed an adequate number of urine samples, we used all data for these Executive Health Clinic patients; thus, our study is subject to selection and confounding bias from this population. Specifically, as our population is comprised of individuals who are fully employed, highly insured, and of high socioeconomic status, our findings may not be generalizable to other populations. Another limitation was the lack of complete smoking data, which was unavailable to the research team. In the absence of a full smoking history, we used a proxy measure that potentially skewed results related to smoking-related risks and affected the study’s reliability and accuracy. Furthermore, without accurate pack-year data, patients may have been incorrectly stratified into intermediate risk rather than high risk, thus diminishing observed differences in disease prevalence and health outcomes and minimizing the true predictive value of the AUA 3- tier module.

Combining intermediate- and high- risk patients into a single group may limit the comprehensiveness and granularity of the risk stratification analysis. Furthermore, our inclusion criteria were based on whether or not patients completed urine collection regardless of known or suspected urologic disease. This approach may have potentially influenced the risk stratification analysis. Finally, our study was primarily descriptive and limited in scope. As a result, we conducted no multivariable adjustments for potential confounders, thus limiting the ability to draw stronger inferences about differences across collection methods and risk groups.

The lack of referrals from primary care providers could have been associated with several external factors, such as the ongoing COVID-19 pandemic and the related health care protocols in place during the final year of the study [29,30]. Significant disruptions in health care services were observed during this period and resulted in a limited ability for providers to offer referrals as a part of routine care, thus leading to underreporting or delays in referral patterns. Finally, due to the relatively small sample size and specific demographic composition, the findings from our risk stratification may not be generalizable to the broader US population.

Future work should continue to investigate the effects of risk stratification on clinical decision-making and access to care, specifically in diverse and underserved populations. Additionally, it is crucial to identify the effect of the new AUA guidelines on physician referral patterns, particularly in relation to the aforementioned gender and race disparities. Investigating the real-world application of these guidelines would provide valuable insight into the barriers and facilitators of implementation on a broader scale.

## Abbreviations

AUA: American Urological Association
HPF: high-powered field
MH: microscopic hematuria
RBC: red blood cell
URBC: red blood cells in urine
